# Editorial: Impact of Special Issue “The Microbial Population of the Gastrointestinal Tract of Animals: Impacts on Host Physiology”

**DOI:** 10.3390/microorganisms12050859

**Published:** 2024-04-25

**Authors:** Jeferson M. Lourenco, Todd R. Callaway

**Affiliations:** Department of Animal and Dairy Science, University of Georgia, Athens, GA 30602, USA; jefao@uga.edu

## Introduction

In recent years, there has been an exponential increase in the number of papers that have investigated the microbiome of animals and humans [1,2,3]. While the relationship between the microbial population and host has been studied for generations, especially in ruminants, unfortunately, much of our understanding of how this relationship is mediated is focused solely around energy (e.g., carbohydrate conversion to volatile fatty acids) and the production of microbial protein. As we have increasingly examined the microbial–host interactions with next-generation sequencing and other current techniques, it has become apparent that the relationships between the microbes and the host are multifaceted and involve many levels of communication [4,5,6]. Ultimately, such interactions can result in distinct combinations of metabolites being produced, with significant impacts on the host [7,8]. Thus, from an animal production perspective, the microbial population can dramatically impact animal health and productivity [3,9,10].

This Special Issue of *Microorganisms* collected eight research articles and three reviews on how the microbial population affects the host animal’s physiology and health. The relationship between the microbial population and host varies based on the animal species; however, across species a common theme emerged, in that we are only scratching the surface in our understanding of the degree to which the native (or introduced) microbial populations can impact the host animal. Several different species of animals were discussed, from teleost fish, shrimp, and gerbils to ruminant animals, as well as humans, highlighting the similarities in the impacts of microbes on the host, from the gut–lung axis to the traditional gut ecosystem, including the impact of diet on the microbial populations. Linkages between the vitamin status and the microbial population in cattle with Johne’s disease were an important point of discussion that brings new considerations to how we view the depth of microbe–host interactions.

The broad selection of topics presented in this collection highlights how far we have come in expanding our knowledge of the microbe–host relationships. However, the more we have learned about these interactions, the more questions have arisen, both in terms of methodology and also about which interactions are most critical in terms of their impact on the host physiology [11,12]. Is it the stimulation of the immune system? Is it the education of the immune system? How important is the production/degradation of vitamins? What role do volatile fatty acids play in host growth? How many catecholamine-like quorum sensing molecules are present that impact the host, and do these differ in each host species? While these gaps in our knowledge are important and are still significant, it is exciting to see the strides that we have made. However, perhaps more importantly, future research into the host–microbiome interaction will improve and elucidate how we feed animals to improve sustainability and reduce the environmental impact while producing animal protein for a growing global human population [13,14].

## References

[1] Seshadri R., Leahy S.C., Attwood G.T., Teh K.H., Lambie S.C., Cookson A.L., Eloe-Fadrosh E.A., Pavlopoulos G.A., Hadjithomas M., Varghese N.J. (2018). Cultivation and sequencing of rumen microbiome members from the Hungate1000 Collection. Nature Biotechnol..

[2] Xiao L., Zhao F. (2023). Microbial transmission, colonisation and succession: From pregnancy to infancy. Gut.

[3] Shalon D., Culver R.N., Grembi J.A., Folz J., Treit P.V., Shi H., Rosenberger F.A., Dethlefsen L., Meng X., Yaffe E. (2023). Profiling the human intestinal environment under physiological conditions. Nature.

[4] Cryan J.F., O’Riordan J.K., Cowan C.S.M., Sandhu K.V., Bastiaanssen T.F.S., Boehme M., Codagnone M.G., Cussotto S., Fulling C., Golubeva A.V. (2019). The Microbiota-Gut-Brain Axis. Physiol. Rev..

[5] Freestone P., Lyte M. (2010). Stress and microbial endocrinology: Prospects for ruminant nutrition. Animal.

[6] Bailey M.T., Dowd S.E., Parry N.M.A., Galley J.D., Schauer D.B., Lyte M. (2010). Stressor exposure disrupts commensal microbial populations in the intestines and leads to increased colonization by *Citrobacter rodentium*. Infect. Immun..

[7] Sasson G., Ben-Shabat S.K., Seroussi E., Doron-Faigenboim A., Shterzer N., Yaacoby S., Miller M.E.B., White B.A., Halperin E., Mizrahi I. (2017). Heritable bovine rumen bacteria are phylogenetically related and correlated with the cow’s capacity to harvest energy from its feed. mBio.

[8] Shabat S.K.B., Sasson G., Doron-Faigenboim A., Durman T., Yaacoby S., Miller M.E.B., White B.A., Shterzer N., Mizrahi I. (2016). Specific microbiome-dependent mechanisms underlie the energy harvest efficiency of ruminants. ISME J..

[9] Sanders D.J., Inniss S., Sebepos-Rogers G., Rahman F.Z., Smith A.M. (2021). The role of the microbiome in gastrointestinal inflammation. Biosci. Rep..

[10] Mayorgas A., Dotti I., Salas A. (2021). Microbial metabolites, postbiotics, and intestinal epithelial function. Mol. Nutr. Food Res..

[11] Adjei-Fremah S., Ekwemalor K., Asiamah E., Ismail H., Ibrahim S., Worku M. (2017). Effect of probiotic supplementation on growth and global gene expression in dairy cows. J. Appl. Anim. Res..

[12] Ohene-Adjei S., Teather R.M., Ivan M., Forster R.J. (2007). Postinoculation protozoan establishment and association patterns of methanogenic archaea in the ovine rumen. Appl. Environ. Microbiol..

[13] Wallace R.J., Sasson G., Garnsworthy P.C., Tapio I., Gregson E., Bani P., Huhtanen P., Bayat A.R., Strozzi F., Biscarini F. (2019). A heritable subset of the core rumen microbiome dictates dairy cow productivity and emissions. Sci. Adv..

[14] De Vrieze J., De Mulder T., Matassa S., Zhou J., Angenent L.T., Boon N., Verstraete W. (2020). Stochasticity in microbiology: Managing unpredictability to reach the Sustainable Development Goals. Microb. Biotechnol..

